# Presymptomatic white matter integrity loss in familial frontotemporal dementia in the GENFI cohort: A cross‐sectional diffusion tensor imaging study

**DOI:** 10.1002/acn3.601

**Published:** 2018-07-11

**Authors:** Lize C. Jiskoot, Martina Bocchetta, Jennifer M. Nicholas, David M. Cash, David Thomas, Marc Modat, Sebastien Ourselin, Serge A.R.B. Rombouts, Elise G.P. Dopper, Lieke H. Meeter, Jessica L. Panman, Rick van Minkelen, Emma L. van der Ende, Laura Donker Kaat, Yolande A.L. Pijnenburg, Barbara Borroni, Daniela Galimberti, Mario Masellis, Maria Carmela Tartaglia, James Rowe, Caroline Graff, Fabrizio Tagliavini, Giovanni B. Frisoni, Robert Laforce, Elizabeth Finger, Alexandre de Mendonça, Sandro Sorbi, on behalf of the Genetic Frontotemporal dementia Initiative (GENFI), Christin Andersson, Silvana Archetti, Andrea Arighi, Luisa Benussi, Sandra Black, Maura Cosseddu, Marie Fallström, Carlos Ferreira, Chiara Fenoglio, Nick Fox, Morris Freedman, Giorgio Fumagalli, Stefano Gazzina, Roberta Ghidoni, Marina Grisoli, Vesna Jelic, Ron Keren, Gemma Lombardi, Carolina Maruta, Simon Mead, Benedetta Nacmias, Linn Öijerstedt, Alessandro Padovani, Michela Pievani, Cristina Polito, Enrico Premi, Sara Prioni, Rosa Rademakers, Ekaterina Rogaeva, Giacomina Rossi, Martin Rossor, Elio Scarpini, David Tang‐Wai, Hakan Thonberg, Pietro Tiraboschi, Ana Verdelho, Jason Warren, Janne M. Papma, John C. van Swieten, Jonathan D. Rohrer

**Affiliations:** ^1^ Department of Neurology Erasmus Medical Center Rotterdam the Netherlands; ^2^ Dementia Research Center University College London London United Kingdom; ^3^ Department of Radiology Leiden University Medical Center Leiden the Netherlands; ^4^ Department of Medical Statistics London School of Hygiene & Tropical Medicine London United Kingdom; ^5^ Centre for Medical Image Computing (CMIC) University College London London United Kingdom; ^6^ Institute of Psychology Leiden University Leiden the Netherlands; ^7^ Leiden Institute for Brain and Cognition Leiden University Leiden the Netherlands; ^8^ Department of Clinical Genetics Erasmus Medical Center Rotterdam the Netherlands; ^9^ Department of Clinical Genetics Leiden University Medical Center Leiden the Netherlands; ^10^ Department of Neurology Alzheimer Center Neuroscience Campus Amsterdam Amsterdam the Netherlands; ^11^ Neurology Unit Department of Clinical and Experimental Sciences University of Brescia Brescia Italy; ^12^ Department of Pathophysiology and Transplantation Dino Ferrari Center University of Milan Fondazione Ca` Granda IRCCS Ospedale Maggiore Policlinico Milan Italy; ^13^ LC Campbell Cognitive Neurology Research Unit Sunnybrook Research Institute Toronto Ontario Canada; ^14^ Tanz Centre for Research in Neurodegenerative Diseases University of Toronto Toronto Ontario Canada; ^15^ Department of Clinical Neurosciences University of Cambridge Cambridge United Kingdom; ^16^ Department of Geriatric Medicine Karolinska University Hospital‐Huddinge Stockholm Sweden; ^17^ Fondazione Istituto di Ricovero e Cura a Carattere Scientifico Istituto Neurologica Carlo Besta Milan Italy; ^18^ Istituto di Ricovero e Cura a Carattere Scientifico (IRCCS) Istituto Centro San Giovanni di Dio Fatebenefratelli Brescia Italy; ^19^ Memory Clinic LANVIE‐Laboratory of Neuroimaging of Aging University Hospitals University of Geneva Geneva Switzerland; ^20^ Clinique Interdisciplinaire de Mémoire Département des Sciences Neurologiques Université Laval Québec Quebec Canada; ^21^ Department of Clinical Neurological Sciences University of Western Ontario Toronto Ontario Canada; ^22^ Faculty of Medicine University of Lisbon Lisbon Portugal; ^23^ Department of NEUROFARBA University of Florence Florence Italy; ^24^ Istituto di Ricovero e Cura a Carattere Scientifico (IRCCS) “Don Gnocchi” Florence Italy; ^25^ Department of Clinical Genetics VU Medical Center Amsterdam the Netherlands

## Abstract

**Objective:**

We aimed to investigate mutation‐specific white matter (WM) integrity changes in presymptomatic and symptomatic mutation carriers of the *C9orf72*,*MAPT*, and *GRN* mutations by use of diffusion‐weighted imaging within the Genetic Frontotemporal dementia Initiative (GENFI) study.

**Methods:**

One hundred and forty mutation carriers (54 *C9orf72*, 30 *MAPT*, 56 *GRN*), 104 presymptomatic and 36 symptomatic, and 115 noncarriers underwent 3T diffusion tensor imaging. Linear mixed effects models were used to examine the association between diffusion parameters and years from estimated symptom onset in *C9orf72*,*MAPT*, and *GRN* mutation carriers versus noncarriers. Post hoc analyses were performed on presymptomatic mutation carriers only, as well as left–right asymmetry analyses on *GRN* mutation carriers versus noncarriers.

**Results:**

Diffusion changes in *C9orf72* mutation carriers are present significantly earlier than both *MAPT* and *GRN* mutation carriers – characteristically in the posterior thalamic radiation and more posteriorly located tracts (e.g., splenium of the corpus callosum, posterior corona radiata), as early as 30 years before estimated symptom onset. *MAPT* mutation carriers showed early involvement of the uncinate fasciculus and cingulum, sparing the internal capsule, whereas involvement of the anterior and posterior internal capsule was found in *GRN*. Restricting analyses to presymptomatic mutation carriers only, similar – albeit less extensive – patterns were found: posteriorly located WM tracts (e.g., posterior thalamic radiation, splenium of the corpus callosum, posterior corona radiata) in presymptomatic *C9orf72*, the uncinate fasciculus in presymptomatic *MAPT*, and the internal capsule (anterior and posterior limbs) in presymptomatic *GRN* mutation carriers. In *GRN*, most tracts showed significant left–right differences in one or more diffusion parameter, with the most consistent results being found in the UF, EC, RPIC, and ALIC.

**Interpretation:**

This study demonstrates the presence of early and widespread WM integrity loss in presymptomatic FTD, and suggests a clear genotypic “fingerprint.” Our findings corroborate the notion of FTD as a network‐based disease, where changes in connectivity are some of the earliest detectable features, and identify diffusion tensor imaging as a potential neuroimaging biomarker for disease‐tracking and ‐staging in presymptomatic to early‐stage familial FTD.

## Introduction

Genetic FTD with an autosomal dominant inheritance pattern has a heterogeneous clinical profile, including behavioral variant FTD (bvFTD) and primary progressive aphasia (PPA). The C*hromosome 9 open reading frame 72* (*C9orf72*) repeat expansion, and mutations in the *microtubule‐associated protein tau* (*MAPT*) and *progranulin* (*GRN*) genes are the three most common causes of familial FTD.^1^, ^2^, ^3^ At‐risk subjects within the presymptomatic stage allow a unique time‐window into the earliest disease stages of FTD, important for diagnostic improvement and the development of robust and sensitive biomarkers.^4^, ^5^ The Genetic Frontotemporal dementia Initiative (GENFI) is a longitudinal cohort study of familial FTD across Europe and Canada, investigating carriers of the *C9orf72, MAPT,* or *GRN* mutations and their healthy first‐degree relatives. Cross‐sectional analyses on volumetric MR images in GENFI demonstrated frontotemporal gray matter (GM) volume loss from 10 years before estimated symptom onset, confirming that the disease process precedes the clinical onset by several years in familial FTD.^6^

White matter (WM) alterations, as measured by diffusion tensor imaging (DTI) are found to be early and widespread in the symptomatic phase of FTD, extending beyond the zones of GM atrophy,^7^, ^8^, ^9^ with distinct profiles in clinical and genetic subtypes.^7^, ^10^, ^11^, ^12^, ^13^, ^14^ The pattern of WM integrity loss includes the uncinate fasciculus (UF), cingulum, (anterior) corpus callosum, fornix, superior and inferior longitudinal fasciculi, thalamic radiation, and corona radiata.^7^, ^12^, ^14^, ^15^, ^16^ Also, previous studies in presymptomatic FTD caused by *GRN* and *MAPT* mutations demonstrated, respectively, lower integrity of the UF,^17^, ^18^ and inferior frontooccipital fasciculus,^17^ whereas studies into presymptomatic *C9orf72* have shown more inconsistent results.^19^, ^20^, ^21^ This underlines that, although a promising candidate, larger studies are needed in order to validate DTI as a neuroimaging biomarker for presymptomatic FTD.

In this study, we compared baseline DTI parameters between mutation carriers and noncarriers in families with autosomal dominant FTD caused by *C9orf72*,* MAPT*, and *GRN* mutations within the GENFI consortium.^6^ We hypothesized that the three different pathogenic groups have distinct profiles, with increasing WM integrity loss when moving from the presymptomatic to early symptomatic stage.

## Methods

### Participants

**Figure 1:**
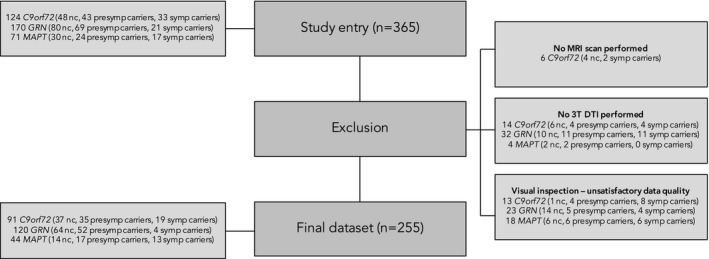
Overview of participant in‐ and exclusion. A total of 365 subjects were eligible for study participation. Six subjects did not undergo MRI scanning, and were therefore excluded. Only 3T scans were considered; therefore, 1.5T (*n* = 34) scans were first excluded from further analysis. Visual quality control of the images resulted in exclusion of another 54 images, leading to a final dataset of 255 subjects (115 noncarriers (“nc”), 104 presymptomatic mutation carriers, 36 symptomatic carriers.

Within the second GENFI data freeze,^6^ 365 participants from genetically confirmed FTD families with either a *C9orf72* repeat expansion, *MAPT*, or *GRN* pathogenic mutation were recruited from 13 research centers between January 30, 2012 and May 4, 2015. Six participants did not have MR imaging performed, and were therefore excluded. To improve data homogeneity, we excluded images from 1.5T scanners (*n* = 50). All images were subjected to strict visual quality control, which led to 54 participants being excluded from further analysis, mainly due to motion and artifacts. The final sample consisted of 255 subjects, of which 140 were mutation carriers (54 *C9orf72*, 30 *MAPT*, 56 *GRN*) and 115 were non‐carriers (see Fig. 1 for the sample flowchart).

### Standard protocol approvals, registrations, and patient consents

Written informed consent was obtained from all participants at study enrolment. The study was approved by the local Medical and Ethical Review committees at each research site. DNA genotyping was performed locally at each research site. We defined a pathogenic repeat expansion in *C9orf72* as more than 30 repeats.^22^ If presymptomatic participants had not undergone predictive testing, the clinical investigators were blinded to their genetic status.

### Clinical assessment

All participants underwent a standardized clinical assessment consisting of a medical and family history, neurological examination, neuropsychological testing, and MR imaging of the brain. We determined clinical status according to established diagnostic criteria,^23^, ^24^ based on this assessment and information from a structured interview with knowledgeable informants. The interview consisted of questions regarding behavioral, neuropsychiatric, cognitive, (instrumental) activities of daily living, motor, and autonomic symptoms. Furthermore, we quantitatively measured functional and/or behavioral changes by means of the Cambridge Behavioural Inventory Revised (CBI‐R).^25^ Global cognition was assessed by means of the Mini‐Mental State Examination (MMSE).^26^

### DTI acquisition and (pre)processing

We performed 3T diffusion‐weighted and volumetric T1‐weighted MRI. Scanning was performed on MRI scanners from five vendors, see Data S2 for an overview of the number of participants and research sites per vendor, and scan parameters. In case diffusion‐weighted images consisted of multiple acquisitions, NifTI files were merged within the FMRIB Software Library (FSL, v5.0.4)^27^; bvec and bval files were concatenated within MATLAB (v2012a). Diffusion‐weighted images were then preprocessed and analyzed using a combination of tools from DTI‐TK (http://dti-tk.sourceforge.net) and NiftyPipe (http://cmictig.cs.ucl.ac.uk/wiki/index.php/NiftyPipe) software packages.^28^ In short, diffusion images were corrected for motion and eddy‐current via an affine coregistration between the diffusion‐weighted image to the average b0 image. Images were also corrected for susceptibility via phase unwrapping.^29^ Diffusion tensor volumes were spatially normalized to a population‐specific tensor template with DTI‐TK.^30^, ^31^ The structural T1‐weighted image was used for reference space. To restrict analyses to brain matter and improve registration, we applied a subject specific binary T1 brain mask image, created by means of the Neuromorphometrics protocol.^32^, ^33^ We extracted diffusion parameters (fractional anisotropy [FA], mean [MD], radial [DR], and axial diffusivity [AxD]) in WM regions‐of‐interest (ROI) from the John Hopkins University (JHU) atlas^34^ using FSL,^27^ and selected the following tracts: uncinate fasciculus (UF); superior longitudinal fasciculus (SLF); cingulum, sagittal stratum; posterior thalamic radiation (PTR); anterior (ACR), posterior (PCR), and superior (SCR) corona radiata; external capsule (EC); anterior (ALIC), and posterior limb (PLIC); and retrolenticular part (RPIC) of the internal capsule; and genu, body, and splenium of the corpus callosum (respectively, gCC, bCC, and sCC). Left and right values were averaged to obtain one value per tract.

### Statistical analysis

Data analysis was carried out using STATA (version 14.2; College Station, TX: StataCorp LP), with the significance level set at *P* < 0.05 (two‐tailed) across all comparisons. Each participant's age at baseline was subtracted from the average age at onset of symptoms in their family to estimate the years to estimated symptom onset (EYO).^6^ EYO, and not actual onset age, was also used in symptomatic mutation carriers in order to provide a common time scale for the analysis. Age at baseline, estimated age at symptom onset, years from estimated symptom onset, and years of education were compared between groups by means of linear regression analyses. Logistic regression was used to investigate differences in sex, and scores on the CBI‐R and MMSE. Robust standard errors were used to account for clustering by family. We used linear mixed effects models to examine whether the association between diffusion parameters and EYO differed between each mutation carrier group (*C9orf72*,* MAPT*, and *GRN*) and noncarriers. A random intercept for family was included, allowing diffusion parameters to be correlated between members of the same family rather than assuming independence. All analyses were adjusted for sex and research site. To allow for nonlinear change in each diffusion parameter, we used a restricted cubic spline^35^ for EYO. The spline terms for EYO were included as predictors in the model, along with the interactions between each spline term for EYO and indicator variables for mutation carrier group (*C9orf72*,* MAPT*, and *GRN*). The spline modeling approach was chosen to allow a complex pattern of association with EYO, for example, FA might increase and then later on become lower. The knots were placed at ‐20, −5, and +7 years relative to expected onset to ensure that each group had a least five participants before the last knot point and after the final knot point, with the middle point splitting the remaining participants into groups of approximately equal sizes. Post hoc, we reran the abovementioned analyses in presymptomatic mutation carriers only (with knots at −20, −10, and 0 EYO). We also investigated left–right differences in *GRN* by calculating the asymmetry in each WM tract as absolute difference in diffusion parameter (left–right)/mean diffusion parameter. As asymmetry values were skewed, we modeled them as a natural log (ln(Asymmetry) – but for easier interpretation, we presented the results after exponentiation giving geometric means and ratios of geometric means between groups. As with previous analyses, we used a mixed effect model allowing for clustering by family and with spline terms for years to estimated symptom onset (knots at −20, −5, and +7 EYO).

For each model, we conducted a hypothesis test of whether the mean value of the diffusion parameter differed between each mutation carrier group (*C9orf72*,* MAPT*, and *GRN*) compared to noncarriers. This was a joint Wald test of the indicator variable for the mutation carrier group of interest and its interactions with the spline terms for EYO. Therefore, for the primary analysis, 60 tests were conducted to compare each mutation carrier group to noncarriers (15 tracts * 4 diffusion parameters). No formal correction was made for multiple comparisons, as diffusion measures were not independent. From each model, we also predicted the mean value of the diffusion parameter for each group, and the differences between each mutation carrier group and noncarriers every year between 30 years before estimated onset and 10 years after estimated onset. We conducted a sensitivity analysis to examine the impact of outliers on the findings on association between diffusion parameters and EYO. The linear mixed effects model described above was repeated for each diffusion parameter in each tract after excluding any participants with model residuals more than three standard deviations away from the predicted mean in the initial analysis.

## Results

### Demographic and clinical data

Demographic and clinical data are shown in Table 1. DNA genotyping assigned participants either to the mutation carrier (*n* = 140; 54 from *C9orf72* families, 30 from *MAPT* families, and 56 from *GRN* families) or noncarrier (*n* = 115; 37 *C9orf72*, 14 *MAPT*, 64 *GRN*) group. Hundred and four participants were presymptomatic (17 *MAPT*, 52 *GRN*, 35 *C9orf72*), and 36 were symptomatic (19 *C9orf72*, 13 *MAPT*, 4 *GRN*). The estimated age at onset was lower in *MAPT* mutation carriers than both *GRN* and *C9orf72* mutation carriers (both *P* < 0.001). All three mutation carrier groups had significantly lower MMSE scores than noncarriers *(C9orf72 P* < 0.001, *GRN P* = 0.006, *MAPT P* = 0.004). CBI‐R scores were significantly higher in *MAPT* and *C9orf72* mutation carriers compared to noncarriers (both *P* < 0.001), and compared to *GRN* mutation carriers (*MAPT P* = 0.002, *C9orf72 P* = 0.003). There were no significant differences regarding sex, age, years from estimated symptom onset, or education. Table 2 provides an overview of the distribution of symptomatic and presymptomatic mutation carriers and noncarriers across EYO. There was one mutation carrier (*C9orf72*) who became symptomatic before their estimated onset age (between −10 and −5 EYO). Nineteen presymptomatic mutation carriers (4 *C9orf72*, 3 *MAPT*, 12 *GRN*) were past their estimated onset age.

**Table 1:** Demographic and clinical data

	Mutation carriers (*n* = 140)	Noncarriers (*n* = 115)	*P‐*value
Female	75 (53.6)	73 (63.5)	0.100
Age (years)	50.1 ± 12.9	49.4 ± 13.3	0.682
Mutated gene			
*C9orf72*	54 (38.6)	37 (32.3)	–
*MAPT*	30 (21.4)	14 (12.2)	–
*GRN*	56 (40.0)	64 (55.7)	–
Clinical status			
Presymptomatic	104 (74.3)	115 (100)	–
Symptomatic	36 (25.7)	0 (0)	–
Estimated age at onset	57.2 ± 6.5	59.3 ± 7.1	0.066
Years from estimated onset	−7.1 ± 12.6	−9.9 ± 14.4	0.193
Education (years)	13.8 ± 3.2	14.0 ± 3.2	0.637
MMSE	28.1 ± 2.8	29.3 ± 1.0	<0.001
CBI‐R	19.7 ± 33.0	3.1 ± 5.4	<0.001

Mutation carriers	*C9orf72*	*MAPT*	*GRN*	*P‐*value^a^
Female	27 (50)	14 (46.7)	34 (60.7)	0.254
Age	50.6 ± 14.0	47.4 ± 12.7	51.0 ± 11.8	0.549
Clinical status				
Presymptomatic	35 (33.7)	17 (16.3)	52 (50)	–
Symptomatic	19 (52.8)	13 (36.1)	4 (11.1)	–
Estimated age at onset	58.3 ± 6.9	51.0 ± 6.0	59.5 ± 3.9	<0.001
Years from estimated onset	−7.7 ± 13.7	−3.6 ± 12.0	−8.5 ± 11.6	0.244
Education (years)	14.0 ± 3.1	13.0 ± 4.0	14.2 ± 2.9	0.668
MMSE	27.8 ± 2.9	27.8 ± 3.7	28.6 ± 2.0	<0.001
CBI‐R	26.3 ± 36.5	33.2 ± 40.0	6.1 ± 16.8	<0.001

Values indicate: count (percentage) or mean ± standard deviation.

*C9orf72*, chromosome 9 open reading frame 72; *MAPT*, microtubule‐associated protein tau; *GRN*, progranulin; MMSE, Mini‐Mental State Examination; CBI‐R, Cambridge Behavioural Inventory – Revised.

a Represents overall *P*‐value for comparison of noncarriers, *C9orf72*,* MAPT*, and *GRN* mutation carriers.

**Table 2:** Distribution of *C9orf72*,* GRN*, and *MAPT* symptomatic mutation carriers, presymptomatic mutation carriers, and noncarriers across estimated years to onset

Mutation	EYO								
	<−25	−25 to −20	−20 to −15	−15 to −10	−10 to −5	−5 to 0	0 to +5	+5 to +10	+10
*C9orf72*									
Symptomatic	0	0	0	0	1	0	5	11	2
Presymptomatic	6	4	8	8	2	3	1	2	1
Noncarriers	7	4	4	5	4	4	2	4	3
*MAPT*									
Symptomatic	0	0	0	0	0	0	4	7	2
Presymptomatic	1	3	4	2	3	1	2	1	0
Noncarriers	0	1	2	4	2	0	1	2	2
*GRN*									
Symptomatic	0	0	0	0	0	0	4	0	0
Presymptomatic	6	4	5	7	9	9	4	8	0
Noncarriers	10	5	8	1	9	12	12	5	2

EYO, estimated years to symptom onset; *C9orf72*, chromosome 9 open reading frame 72; *MAPT*, microtubule‐associated protein tau; *GRN*, progranulin.

#### *C9orf72* mutation carriers

**Figure 2:**
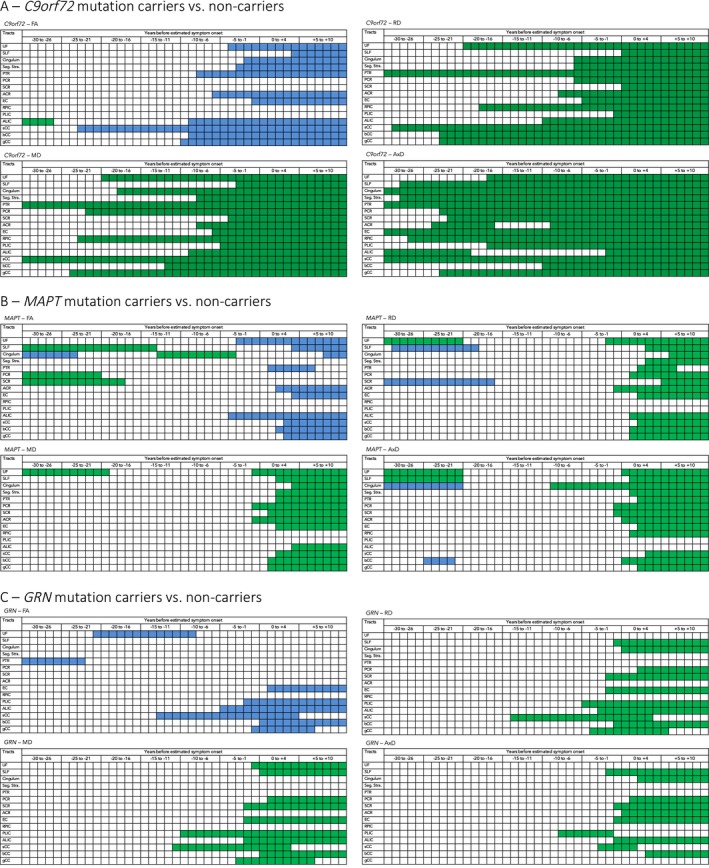
Gene‐specific differences in WM integrity between mutation carriers and noncarriers between minus 30 years before estimated onset until 10 years postestimated onset. Schematic overview of mean diffusion differences between noncarriers and *C9orf72* (A), *MAPT* (B) and *GRN* mutation carriers (C) between minus 30 years before estimated symptom onset and plus 10 years after estimated onset (*x*‐axis), each row represents a different WM tract (*y*‐axis). Blue = where the difference between mutation carriers and noncarriers is negative; green = where the difference between mutation carriers and noncarriers is positive. NB: for FA, blue represents lower FA (=lower WM integrity) in mutation carriers than in noncarriers; for MD, RD, and AxD, green represents higher parameters (=lower WM integrity) in mutation carriers than in noncarriers. UF, uncinate fasciculus; SLF, superior longitudinal fasciculus; Sag.Stra., sagittal stratum; PTR, posterior thalamic radiation; PCR, posterior corona radiata; SCR, superior corona radiata; ACR, anterior corona radiata; EC, external capsule; RPIC, retrolenticular part of the internal capsule; PLIC, posterior limb of the internal capsule; ALIC, anterior limb of the internal capsule; sCC, splenium of the corpus callosum; bCC, body of the corpus callosum; gCC, genu of the corpus callosum.

Analyses of all symptomatic and presymptomatic *C9orf72* repeat expansion carriers demonstrated significant presymptomatic differences across all WM tracts and diffusion metrics (Table 3, Fig. 2A). The earliest presymptomatic changes – between 30 and 20 years before estimated onset – were seen in the PTR, PCR, RPIC, sCC, and gCC, followed by the UF and cingulum. In the last decade prior to estimated onset, significant differences were also found in the bCC, PLIC, EC, SCR, sagittal stratum, and SLF. Surprisingly, in the ALIC, the diffusivity values in repeat expansion carriers became more similar to those of noncarriers, and did not differ significantly during an intermediate period from respectively 20–10 years before estimated symptom onset. See Data S3 for means, mean differences, and *P*‐values per year before estimated onset. Post hoc analyses on only presymptomatic expansion carriers demonstrated similar – albeit less extensive – patterns of WM integrity loss, with the earliest and most consistent differences found in the posterior WM tracts, for example, PTR, sCC, PCR (Data S4A). In the late presymptomatic stage the internal capsule (RPIC, ALIC, PLIC) also became involved (Data S4A).

**Table 3:**
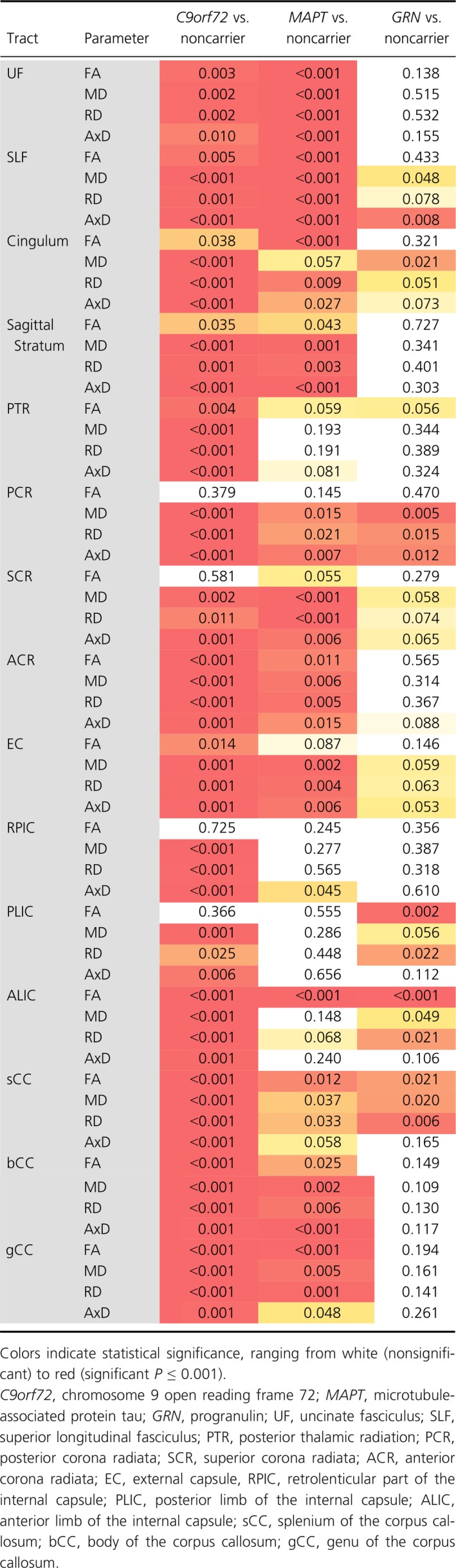
*P*‐values for difference in diffusion parameters between mutation carriers and noncarriers

#### *MAPT* mutation carriers

Analyses of all symptomatic and presymptomatic *MAPT* mutation carriers had significant differences from noncarriers across several WM tracts (Table 3, Fig. 2B). There was very early involvement of the UF: higher diffusivity was found between 30 and 20 years before estimated symptom onset, and again from 3 years before estimated symptom onset. Somewhat inconsistent findings were demonstrated for the SLF, cingulum, and SCR: all three tracts had a presymptomatic time‐window in which FA was increased, while diffusivity values were decreased. After estimated symptom onset, mutation carriers also had changes in the PCR, ACR, sagittal stratum, PTR, EC, and corpus callosum (gCC, bCC, and sCC). There was weaker evidence for differences in the RPIC, PLIC, and ALIC, and even 10 years postonset values did not show consistent differences compared with noncarriers. See Data S3 for means, mean differences, and *P*‐values per year before estimated onset. Post hoc analyses on presymptomatic mutation carriers only confirm the early involvement of the UF: AxD changes are found between −30 and −24 years before estimated symptom onset, followed by changes in FA, MD, and RD shortly before or around estimated symptom onset (Data S4B). Furthermore, early presymptomatic changes were also found in the cingulum, SLF, and SCR. After estimated symptom onset, the internal capsule (ALIC and PLIC) also demonstrated diffusivity changes (Data S4B).

#### *GRN* mutation carriers

Analyses of all symptomatic and presymptomatic *GRN* mutation carriers had significant differences from noncarriers across a relatively limited number of WM tracts (Table 3, Fig 2C). The strongest evidence for differences were in the PLIC, ALIC, PCR, SCR, sCC, SLF, and cingulum. The most consistent presymptomatic WM integrity changes were in the ALIC and PLIC, which showed significant differences from noncarriers from 10 years before estimated onset. Early presymptomatic changes were also found in the sCC, but differences only remained significant up to 4 years postonset. The SLF and SCR showed differences from 1 to 2 years before estimated onset, followed by the cingulum and PCR only after estimated onset. The overall test comparing *GRN* mutation carriers to noncarriers did not show evidence for WM integrity changes in UF, sagittal stratum, PTR, ACR, RPIC, bCC, and gCC. It was particularly notable that even 10 years after estimated onset, the diffusivity values of the sagittal stratum, RPIC, ACR, and PTR were not significantly different between mutation carriers and noncarriers. See Data S3 for means, mean differences, and *P*‐values per year before estimated onset. Post hoc analyses on only presymptomatic mutation carriers showed consistent diffusivity changes in the internal capsule (ALIC and PLIC) alone (Data S4C).

We additionally investigated left–right differences between *GRN* mutation carriers and noncarriers. In most tracts significant left–right differences were found between groups in one or more diffusion parameters (Data S5). The most consistent results were found in the UF, EC, RPIC, and ALIC (Data S5). Asymmetry in the UF was mostly present in the early presymptomatic stage (−30 to +1 EYO), while the EC, RPIC, and ALIC demonstrated asymmetry across the entire EYO range for different diffusion parameters (Data S6). Interestingly, the four tracts demonstrated different patterns over time (Data S6). The UF showed less asymmetry with disease progression, while a sharp postonset increase was seen for the ALIC. In the EC a *U*‐shape pattern was visible, with first a decrease in asymmetry in the early presymptomatic stage, followed by an increase from around −5 EYO. The RPIC demonstrated an inverse *U*‐shape, with first more asymmetry in the early presymptomatic stage, followed by a decrease from around −5 EYO.

### Sensitivity analysis

The number of outliers that were excluded for the sensitivity analysis depended on the tract and DTI parameter, with a maximum of five outliers. Findings were comparable once these outliers were excluded. For the *C9orf72* mutation carriers significant differences with noncarriers were apparent in all WM tracts. These differences remained apparent up to 30 years before estimated symptom onset. In *MAPT* mutation carriers there remained differences in the WM tracts that were previously identified and the same pattern remained with early involvement of the UF. For *GRN* mutation carriers consistent differences were still detected in the same WM tracts with the earliest differences seen in the ALIC and PLIC.

## Discussion

This study describes WM integrity changes by means of DTI in mutation carriers and noncarriers from families with autosomal dominant FTD due to mutations in *C9orf72*,* MAPT*, and *GRN*, within the GENFI consortium. Early WM involvement was found in mutation carriers, with specific genetic patterns for the *C9orf72*,* MAPT*, and *GRN* mutations. Our study suggests spreading WM integrity loss toward symptom onset, highlighting the value of DTI as disease‐tracking and ‐staging biomarker in familial FTD.

The pattern of WM integrity changes in the early presymptomatic stage shows large resemblance to the regions known to be affected in both familial^7^, ^12^, ^15^, ^16^ and sporadic^11^, ^36^, ^37^, ^38^, ^39^ symptomatic FTD. Furthermore, although the cohort was somewhat different, the damage to the WM seems to be earlier and more widespread than the GM volume loss found earlier in GENFI,^6^ a finding consistent with previous work in presymptomatic familial^17^, ^18^ and sporadic FTD.^11^, ^36^, ^37^, ^38^, ^39^ More WM tracts appear to be involved in this study compared to previous studies of presymptomatic familial FTD.^17^, ^18^, ^19^, ^20^, ^21^ An explanation for this more extensive involvement may be sought in our larger sample size (more power to detect small differences, and covering a broader presymptomatic period) and the use of all four diffusion parameters, compared to FA only in previous studies. The additional three diffusivity parameters appeared to be more sensitive than FA, and may provide more accurate measures of the effect and extent of the WM integrity changes in the presymptomatic phase.

The most interesting findings are the gene‐specific “fingerprints” of WM integrity loss in *C9orf72*,* MAPT*, and *GRN* mutation carriers. Restricting our analyses to presymptomatic mutation carriers confirmed these findings. In *C9orf72*, specifically the more posteriorly located tracts, such as the PTR, PCR, and sCC, are affected. The PTR demonstrates the earliest changes already 30 years before estimated onset – suggesting that damage might be present even before that. This is in line with earlier findings in the GENFI cohort showing GM volume loss of the thalamus and posterior cortical areas from 25 years before estimated onset.^6^ The similar pattern and timing of WM pathology seems consistent with the long‐standing and slowly progressive symptomatic changes often seen in this mutation,^40^, ^41^ and coherent with the hypothesis of a developmental origin in *C9orf72*‐associated FTD.^6^ In both *MAPT* and *GRN*, WM changes have been consistently found later than in *C9orf72*. The observation of presymptomatic changes in the UF and cingulum in *MAPT* mutation carriers is consistent with smaller series of presymptomatic^18^ and symptomatic carriers,^7^, ^42^ and congruent with tracts affected in bvFTD, the most common clinical phenotype of *MAPT*.^43^ We could not confirm greater WM damage in the SLF in symptomatic cases with underlying FTD‐tau than FTD‐TDP (e.g., *GRN* or *C9orf72*) found in a previous study,^14^ suggesting that this difference might occur later in the disease process or resembles a phenotypic rather than genotypic origin.^13^, ^14^ We could not explain the remarkable finding of DTI changes into the opposite direction in the SLF, SCR, cingulum between −30 and −16 years before EYO, and larger samples and follow‐up data (longitudinal changes within an individual) are needed to investigate whether the pattern is of pathophysiological or methodological nature. Recent literature provides evidence of WM involvement in *GRN*‐related FTD,^44^, ^45^ though on the contrary in our *GRN* mutation carriers few tracts were affected, and integrity loss was generally closer to estimated symptom onset than early presymptomatic. Previous studies demonstrated lower FA in the UF of presymptomatic *GRN* mutation carriers,^17^, ^18^ and we did find lower FA in the presymptomatic period, but no differences in the symptomatic stage or in other diffusion parameters. One potential explanation for this discrepancy could be the large variation in age at onset within *GRN* families,^46^ making the estimated age at onset less reliable than in the other mutations. Another point for consideration here is the potential masking of effects by taking the mean value per WM tract, given the asymmetric neuroimaging phenotype of *GRN*.^47^

Left–right asymmetry was present in most WM tracts of *GRN* mutation carriers, with the most consistent asymmetry being found in the UF, EC, RPIC, and ALIC. These results not only demonstrate that some tracts are more vulnerable to disproportionate WM integrity loss than others (e.g., no asymmetry in the corona radiata), but also that the development of asymmetry has a different timing and pattern in various WM tracts. In line with previous neuroimaging research, showing more asymmetry with disease progression in symptomatic *GRN* mutation carriers,^48^ we found a sharp increase in asymmetry after estimated symptom onset in the ALIC, whereas the inverse was seen in the UF. Rohrer et al.^6^ found greater whole brain GM asymmetry in presymptomatic *GRN* mutation carriers starting 5 years before estimated symptom onset. Also in our study the −5 EYO seems to be a critical time point in the development of WM asymmetry, with the EC demonstrating more asymmetry and the RPIC showing less asymmetry after −5 EYO. More research using longitudinal data is needed to investigate the development of asymmetry over time in more detail.

The development of sensitive biomarkers for diagnosis, for example, differentiation between clinical, genetic, or pathological subtypes, and staging purposes is one of the main challenges in presymptomatic FTD, as future therapeutic interventions ideally start in the unique time‐window of minimal pathological damage. Although the identification of “upstream” biomarkers is essential for the development of therapeutic trials, the connectivity correlates of FTD pathophysiological processes were thus far unknown for the presymptomatic stage.^13^ Our results demonstrate the potential application of DTI as a future diagnostic and staging biomarker – providing evidence of very early presymptomatic alterations as well as consistent WM integrity loss when moving from the late presymptomatic into the early symptomatic stage. Also, mutation‐specific profiles for *C9orf72*,* MAPT*, and *GRN* suggest the potential of DTI in pathology‐specific clinical trials. In contrast to FA reductions as a measure of WM integrity loss in previous studies,^49^ diffusivity measures (MD, RD, AxD) reflected early WM alterations much more sensitively. This is consistent with a previous study into the clinical subtypes of FTD,^50^ supporting the notion that FA does not capture the full extent of WM pathology, and the four metrics signify different underlying processes with disease progression. As a next step, postmortem studies are needed to increase our understanding of the histopathological representation of WM changes in relationship with markers of demyelination, neuroinflammation, neuronal loss, and underlying pathology. Furthermore, to use DTI in clinical practice, more research is needed on the translation of our group‐based results to the individual patient level. Larger studies are also needed to differentiate pathological subtypes in individual patients.^51^ Lastly, as neurofilament light chain is thought to be a sensitive marker of axonal damage, and therefore could be associated with DTI,^52^ it would be interesting to investigate this biomarker further in this cohort.

Key strengths of our study constitute the large sample of FTD mutation carriers and noncarriers. Our study describes the presymptomatic to early symptomatic stage of familial FTD in a long time trajectory of 40 years, with only a single symptomatic mutation carrier (*C9orf72*) before their estimated onset age. Therefore, the influence of this symptomatic mutation carrier is most likely very minimal. With respect to preprocessing, registration was improved by computing the image similarity on the basis of full tensor images rather than scalar features, in which the algorithm incorporates local fiber orientations as features driving the alignment of individual WM tracts. The use of only 3T images, extensive data control after each preprocessing step, and our sensitivity analysis further ascertained data homogeneity. In the pilot phase of GENFI, more variable DTI acquisition parameters and protocols (e.g., use of field and phase maps) were used, introducing a source of bias to the data. Now in the second phase of GENFI, scan protocols have been fully harmonized, so that from 2015 onwards we are building on a much more consistent dataset. Exploring the involvement of corticospinal tracts, as recent research demonstrated early damage in *C9orf72*‐associated ALS,^19^ bvFTD, and PPA,^50^ would be a very informative next step. Other future directions include the investigation of DTI as a longitudinal neuroimaging biomarker and its potential role in multimodal and composite scores in presymptomatic FTD.

Our study provides evidence of global and gene‐specific WM integrity loss as an early pathological feature of presymptomatic familial FTD, making DTI a promising diagnostic and staging neuroimaging biomarker that in the future could be used in upcoming clinical trials for familial FTD.

## Author Contributions

LCJ drafted the body of the manuscript, tables, and figures. MB contributed to the statistical analyses. JDR contributed to the design of the study, data interpretation, and the writing process. JMN performed the statistical analyses and contributed to the writing process. JMP and JvS contributed to data interpretation and the writing process. RvM, SM, ER, HT, LB, GB, and BN did the genetic analyses. All authors recruited patients, collected data, and contributed by reviewing and editing of the manuscript.

## Conflicts of Interest

The authors report no conflict of interest with respect to the work in the manuscript.

## Supporting information


**Data S1**. GENFI consortium members.

**Data S2**. Overview of MRI scanners and scan parameters.

**Data S3**. Whole‐group and gene‐specific WM diffusion differences between mutation carriers and noncarriers (raw values).

**Data S4**. Gene‐specific differences in WM integrity in presymptomatic mutation carriers only.

**Data S5**. Left–right asymmetry *P*‐values for *GRN* mutation carriers versus noncarriers.

**Data S6**. Ratio values across estimated years to symptom onset in GRN mutation carriers versus noncarriers.
