# Supplementary material for: Presymptomatic white matter integrity loss in familial frontotemporal dementia in the GENFI cohort: A cross‐sectional diffusion tensor imaging study
Source: Ann Clin Transl Neurol. 2018 Jul 11;5(9):1025–36. doi: 10.1002/acn3.601 (PMC6144447; doi:10.1002/acn3.601)
Supplement: Supplementary file 1 — Data S1. GENFI consortium members. [file ACN3-5-1025-s001.docx]

**Supplement 1 – GENFI consortium members**

- Christin Andersson - Department of Clinical Neuroscience, Karolinska Institutet, Stockholm, Sweden
- Silvana Archetti – Biotechnology Laboratory, Department of Diagnostics, Civic Hospital of Brescia, Brescia, Italy
- Andrea Arighi - Neurology Unit, Department of Physiopathology and Transplantation, Fondazione Cà Granda, Istituto di Ricovero e Cura a Carattere Scientifico Ospedale Policlinico, Milan, Italy
- Luisa Benussi - Istituto di Ricovero e Cura a Carattere Scientifico Istituto Centro San Giovanni di Dio Fatebenefratelli, Brescia, Italy
- Sandra Black - LC Campbell Cognitive Neurology Research Unit, Sunnybrook Research Institute, Toronto, Canada
- Maura Cosseddu - Centre of Brain Aging, University of Brescia, Brescia, Italy
- Marie Fallström - Department of Geriatric Medicine, Karolinska University Hospital, Stockholm, Sweden
- Carlos Ferreira -  Instituto Ciências Nucleares Aplicadas à Saúde, Universidade de Coimbra,Coimbra, Portugal
- Chiara Fenoglio - Dept. of Pathophysiology and Transplantation, "Dino Ferrari" Center, University of Milan, Fondazione Cà Granda, IRCCS Ospedale Maggiore Policlinico, Milan, Italy
- Nick Fox – Dementia Research Centre, UCL Institute of Neurology, London, UK
- Morris Freedman - Division of Neurology, Baycrest Centre for Geriatric Care, University of Toronto, Canada
- Giorgio Fumagalli - Neurology Unit, Fondazione Cà Granda, Istituto di Ricovero e Cura a Carattere Scientifico Ospedale Policlinico, Milan, Italy
- Christin Andersson - Department of Clinical Neuroscience, Karolinska Institutet, Stockholm, Sweden
- Silvana Archetti – Biotechnology Laboratory, Department of Diagnostics, Civic Hospital of Brescia, Brescia, Italy
- Andrea Arighi - Neurology Unit, Department of Physiopathology and Transplantation, Fondazione Cà Granda, Istituto di Ricovero e Cura a Carattere Scientifico Ospedale Policlinico, Milan, Italy
- Luisa Benussi - Istituto di Ricovero e Cura a Carattere Scientifico Istituto Centro San Giovanni di Dio Fatebenefratelli, Brescia, Italy
- Sandra Black - LC Campbell Cognitive Neurology Research Unit, Sunnybrook Research Institute, Toronto, Canada
- Maura Cosseddu - Centre of Brain Aging, University of Brescia, Brescia, Italy
- Marie Fallström - Department of Geriatric Medicine, Karolinska University Hospital, Stockholm, Sweden
- Carlos Ferreira -  Instituto Ciências Nucleares Aplicadas à Saúde, Universidade de Coimbra,Coimbra, Portugal
- Chiara Fenoglio - Dept. of Pathophysiology and Transplantation, "Dino Ferrari" Center, University of Milan, Fondazione Cà Granda, IRCCS Ospedale Maggiore Policlinico, Milan, Italy
- Nick Fox – Dementia Research Centre, UCL Institute of Neurology, London, UK
- Morris Freedman - Division of Neurology, Baycrest Centre for Geriatric Care, University of Toronto, Canada
- Giorgio Fumagalli - Neurology Unit, Fondazione Cà Granda, Istituto di Ricovero e Cura a Carattere Scientifico Ospedale Policlinico, Milan, Italy
- Stefano Gazzina - Centre of Brain Aging, Neurology Unit, Department of Clinical and Experimental Sciences, University of Brescia, Brescia, Italy
- Roberta Ghidoni - Istituto di Ricovero e Cura a Carattere Scientifico Istituto Centro San Giovanni di Dio Fatebenefratelli, Brescia, Italy
- Marina Grisoli - Fondazione Istituto di Ricovero e Cura a Carattere Scientifico Istituto Neurologico Carlo Besta, Milano, Italy
- Vesna Jelic - Division of Clinical Geriatrics, Karolinska Institutet, Stockholm, Sweden
- Ron Keren - University Health Network Memory Clinic, Toronto Western Hospital, Toronto, Canada
- Gemma Lombardi - Department of Neuroscience, Psychology, Drug Research and Child Health, University of Florence, Florence, Italy
- Carolina Maruta - Lisbon Faculty of Medicine, Language Research Laboratory, Lisbon, Portugal
- Simon Mead – MRC Prion Unit, Department of Neurodegenerative Disease, UCL Institute of Neurology, UK
- Benedetta Nacmias - Department of Neuroscience, Psychology, Drug Research and Child Health, University of Florence, Florence, Italy
- Linn Öijerstedt - Division of Neurogeriatrics, Karolinska Institutet, Stockholm, Sweden
- Alessandro Padovani - Neurology Unit, Department of Medical and Experimental Sciences, University of Brescia, Brescia, Italy
- Michela Pievani - Istituto di Ricovero e Cura a Carattere Scientifico Istituto Centro San Giovanni di Dio Fatebenefratelli, Brescia, Italy
- Cristina Polito - Department of Clinical Pathophysiology, University of Florence, Florence, Italy
- Enrico Premi - Centre for Ageing Brain and Neurodegenerative Disorders, Neurology Unit, University of Brescia, Brescia, Italy
- Sara Prioni - Fondazione Istituto di Ricovero e Cura a Carattere Scientifico Istituto Neurologico Carlo Besta, Milano, Italy
- Rosa Rademakers - Department of Neurosciences, Mayo Clinic, Jacksonville, Florida
- Veronica Redaelli - Fondazione Istituto di Ricovero e Cura a Carattere Scientifico Istituto Neurologico Carlo Besta, Milano, Italy
- Ekaterina Rogaeva - Tanz Centre for Research in Neurodegenerative Diseases, University of Toronto, Canada
- Giacomina Rossi - Fondazione Istituto di Ricovero e Cura a Carattere Scientifico Istituto Neurologico Carlo Besta, Milano, Italy
- Martin Rossor – Dementia Research Centre, UCL Institute of Neurology, London, UK
- Elio Scarpini - Neurology Unit, Department of Physiopathology and Transplantation, Fondazione Cà Granda, Istituto di Ricovero e Cura a Carattere Scientifico Ospedale Policlinico, Milan, Italy
- David Tang-Wai - University Health Network Memory Clinic, Toronto Western Hospital, Toronto, Canada
- Hakan Thonberg - Center for Alzheimer Research, Division of Neurogeriatrics, Karolinska Institutet, Stockholm, Sweden
- Pietro Tiraboschi - Fondazione Istituto di Ricovero e Cura a Carattere Scientifico Istituto Neurologico Carlo Besta, Milano, Italy
- Ana Verdelho - Department of Neurosciences, Santa Maria Hospital, University of Lisbon, Portugal
- Jason Warren – Dementia Research Centre, UCL Institute of Neurology, London, UK
