# Supplementary material for: Presymptomatic white matter integrity loss in familial frontotemporal dementia in the GENFI cohort: A cross‐sectional diffusion tensor imaging study
Source: Ann Clin Transl Neurol. 2018 Jul 11;5(9):1025–36. doi: 10.1002/acn3.601 (PMC6144447; doi:10.1002/acn3.601)
Supplement: Supplementary file 2 — Data S2. Overview of MRI scanners and scan parameters. [file ACN3-5-1025-s002.docx]

**Supplement 2. Overview of MRI scanners and scan parameters.**

| **Vendor** | **Philips** | **GE** | **Siemens** | **Siemens** | **Siemens** |
| --- | --- | --- | --- | --- | --- |
| **Type** | Achieva | Discovery MR750 | Trio | Allegra | Skyra |
| **Scans in study** | 138 | 20 | 88 | 3 | 6 |
| **Research sites** | 3 | 1 | 5 | 1 | 1 |
| **FM echo time difference (ms)** | 2.46 | 2.60 | 2.46 | 2.46 | 2.46 |
| **Diffusion EPI readout time (ms)** | 32.57 | 28.42 | 34.56 | 35.52 | 34.56 |
| **PE direction** | AP | AP | AP | AP | AP |
| **FOV (mm)** | 240x240 | 240x240 | 240x240 | 240x240 | 240x240 |
| **TE (ms)** | 69 | 91 | 91 | 83 | 91 |
| **TR (ms)** | 7000 | 6900 | 6900 | 6900 | 6900 |
| **Acquisition matrix** | 96x96 | 96x96 | 96x96 | 96x96 | 96x96 |
| **Slice number** | 55 | 48 | 55 | 55 | 55 |
| **Slice thickness (mm)** | 2.5 | 2.9 | 2.5 | 3 | 3 |
| **Number of different directions** | 64 | 64 | 64 | 64 | 64 |
| **B value** | 1000 | 1000 | 1000 | 1000 | 1000 |
| **Repeats** | 2 (1 with 4xb=0,  1 with 5xb=0) | 3 (1 with 4xb=0,  1 with 5xb=0) | 4 (1 with 4xb=0,  1 with 5xb=0) | 5 (1 with 4xb=0,  1 with 5xb=0) | 6 (1 with 4xb=0,  1 with 5xb=0) |

Abbreviations: GE, General Electric; FM, field map; EPI, echo-planar imaging; PE, phase-encoding; AP, anterior-posterior; FOV, field of view; TE, echo time; TR, repetition time.
