# Supplementary material for: Presymptomatic white matter integrity loss in familial frontotemporal dementia in the GENFI cohort: A cross‐sectional diffusion tensor imaging study
Source: Ann Clin Transl Neurol. 2018 Jul 11;5(9):1025–36. doi: 10.1002/acn3.601 (PMC6144447; doi:10.1002/acn3.601)

A – *C9orf72* mutation carriers vs. non-carriers

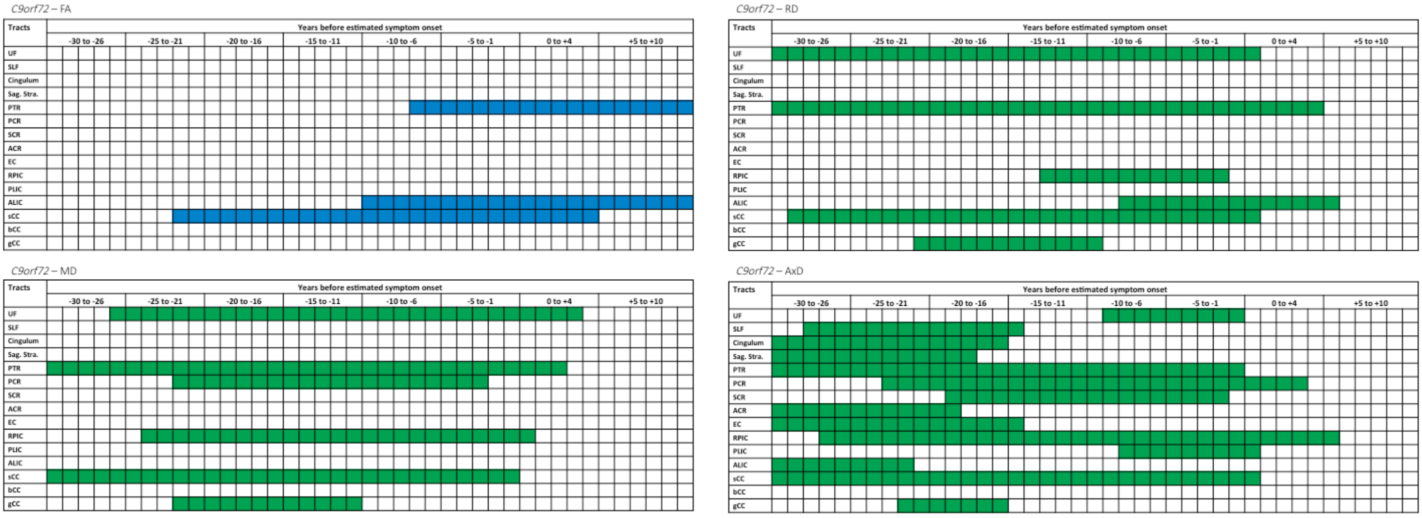

B – *MAPT* mutation carriers vs. non-carriers

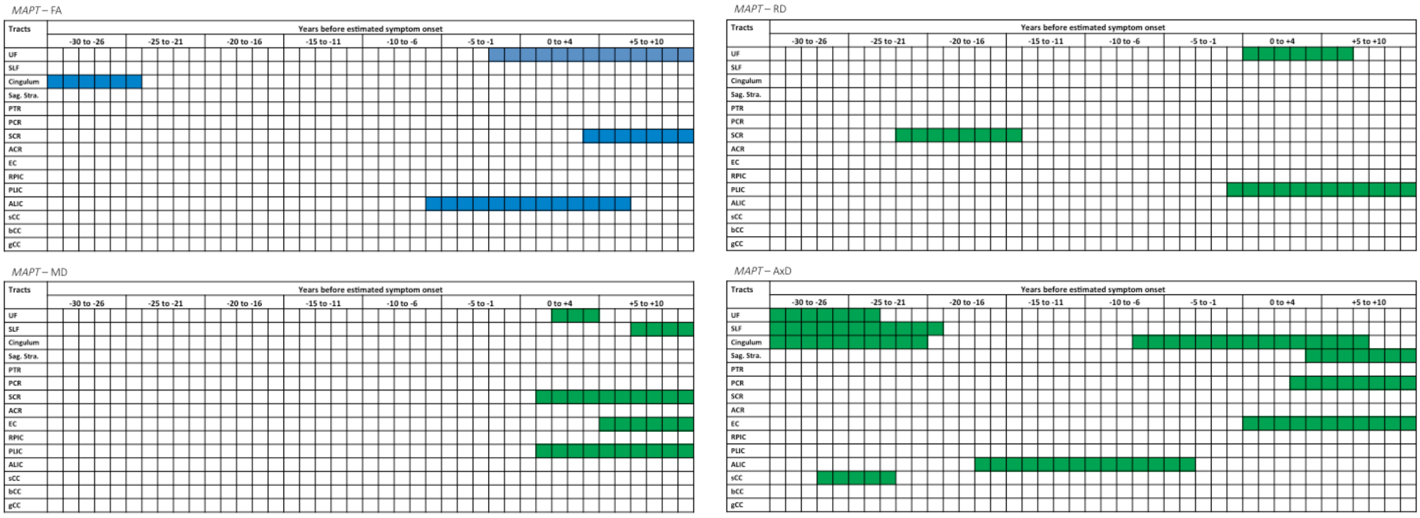

C – *GRN* mutation carriers vs. non-carriers

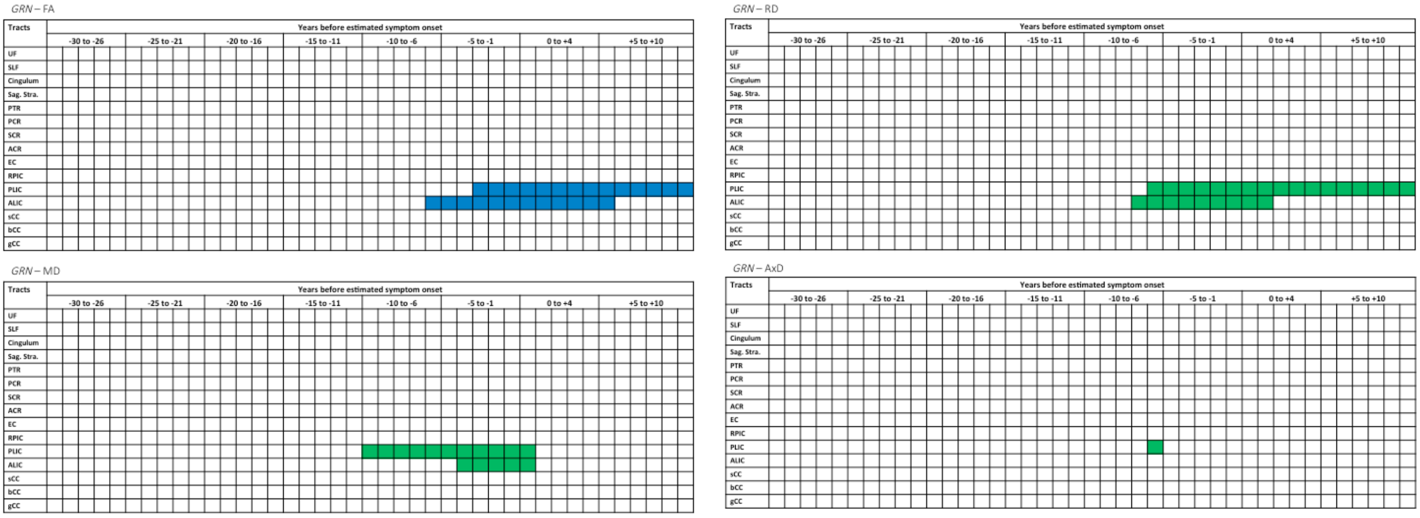

Supplement: Supplementary file 4 — Data S4. Gene‐specific differences in WM integrity in presymptomatic mutation carriers only. [file ACN3-5-1025-s004.pdf]
