# Supplementary material for: Presymptomatic white matter integrity loss in familial frontotemporal dementia in the GENFI cohort: A cross‐sectional diffusion tensor imaging study
Source: Ann Clin Transl Neurol. 2018 Jul 11;5(9):1025–36. doi: 10.1002/acn3.601 (PMC6144447; doi:10.1002/acn3.601)
Supplement: Supplementary file 5 — Data S5. Left–right asymmetry P‐values for GRN mutation carriers versus noncarriers. [file ACN3-5-1025-s005.docx]

**Supplement 5. Left-right asymmetry p-values for *GRN* mutation carriers vs. non-carriers.**

|  | **FA** | **MD** | **RD** | **DA** |
| --- | --- | --- | --- | --- |
| **UF** | **0.028** | **0.016** | **0.028** | 0.186 |
| **SLF** | 0.656 | 0.294 | 0.656 | **0.009** |
| **Cingulum** | **0.004** | 0.689 | **0.004** | **0.045** |
| **Sagittal stratum** | **0.013** | **0.018** | **0.013** | **0.007** |
| **PTR** | 0.747 | 0.174 | 0.747 | 0.948 |
| **PCR** | 0.253 | **0.004** | 0.253 | 0.115 |
| **SCR** | 0.948 | 0.587 | 0.948 | 0.470 |
| **ACR** | **0.049** | 0.112 | **0.049** | 0.080 |
| **EC** | 0.135 | **0.011** | 0.135 | **0.029** |
| **RPIC** | 0.499 | 0.075 | 0.499 | **<0.001** |
| **PLIC** | 0.154 | **0.001** | 0.154 | **0.049** |
| **ALIC** | **0.003** | **0.005** | **0.003** | 0.440 |

Values indicate p-values for *GRN* mutation carriers vs. non-carriers. Abbreviations: *GRN*, progranulin; UF, uncinate fasciculus; SLF, superior longitudinal fasciculus; PTR, posterior thalamic radiation; PCR, posterior corona radiata; SCR, superior corona radiata; ACR, anterior corona radiata; EC, external capsule; RPIC, retrolenticular part of the internal capsule; PLIC, posterior limb of the internal capsule; ALIC, anterior limb of the internal capsule.
