# Supplementary figures and images for: Presymptomatic white matter integrity loss in familial frontotemporal dementia in the GENFI cohort: A cross‐sectional diffusion tensor imaging study
Source: Ann Clin Transl Neurol. 2018 Jul 11;5(9):1025–36. doi: 10.1002/acn3.601 (PMC6144447; doi:10.1002/acn3.601)

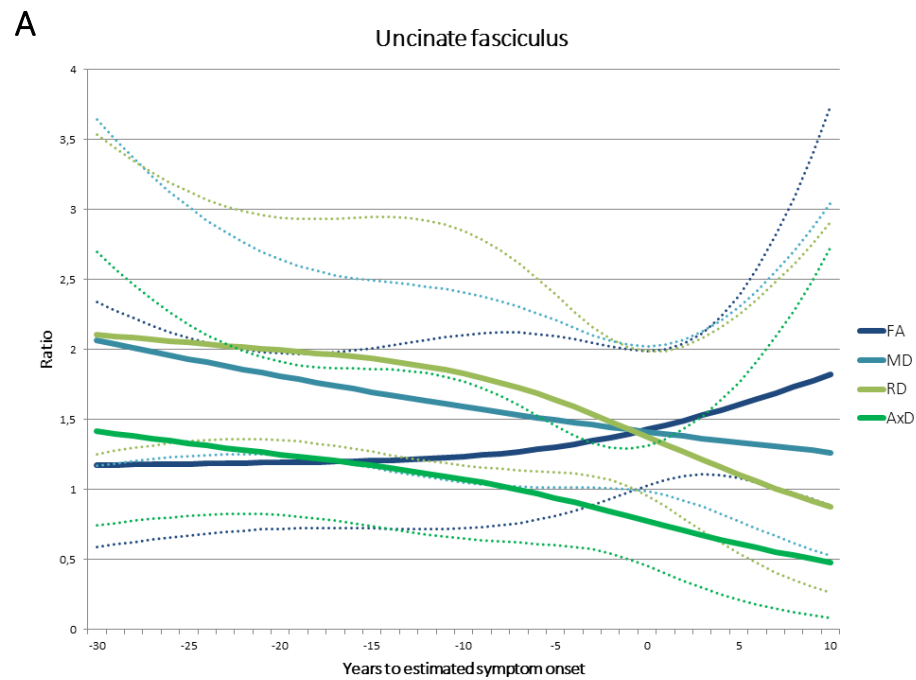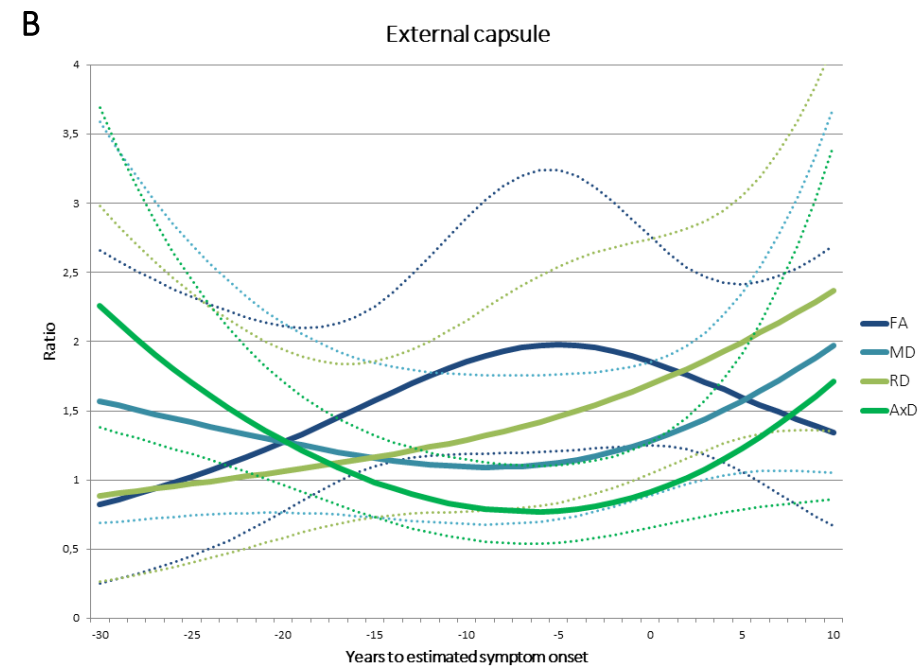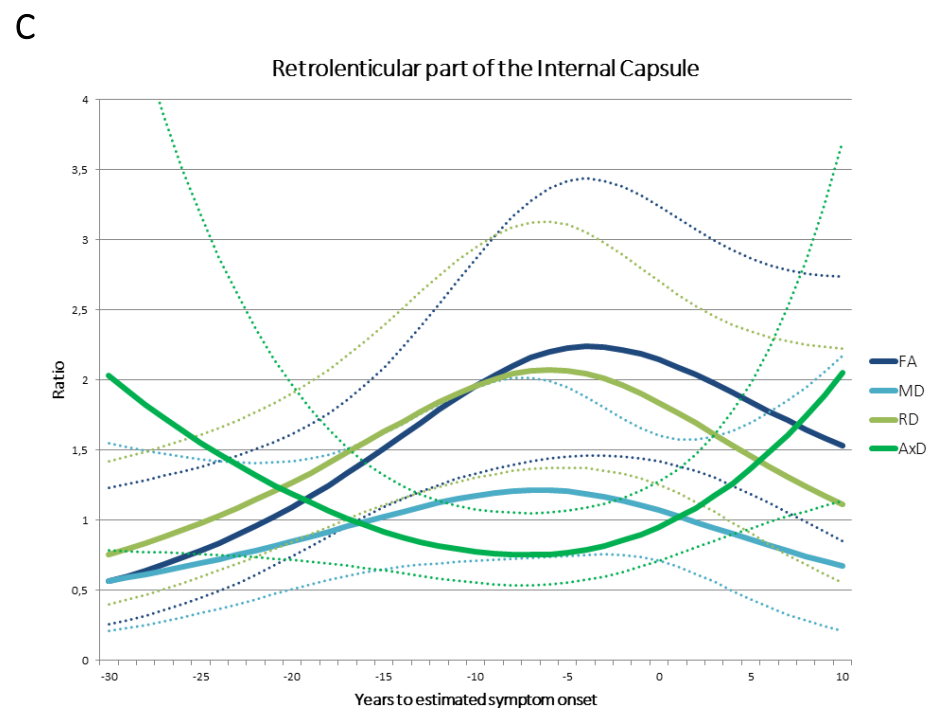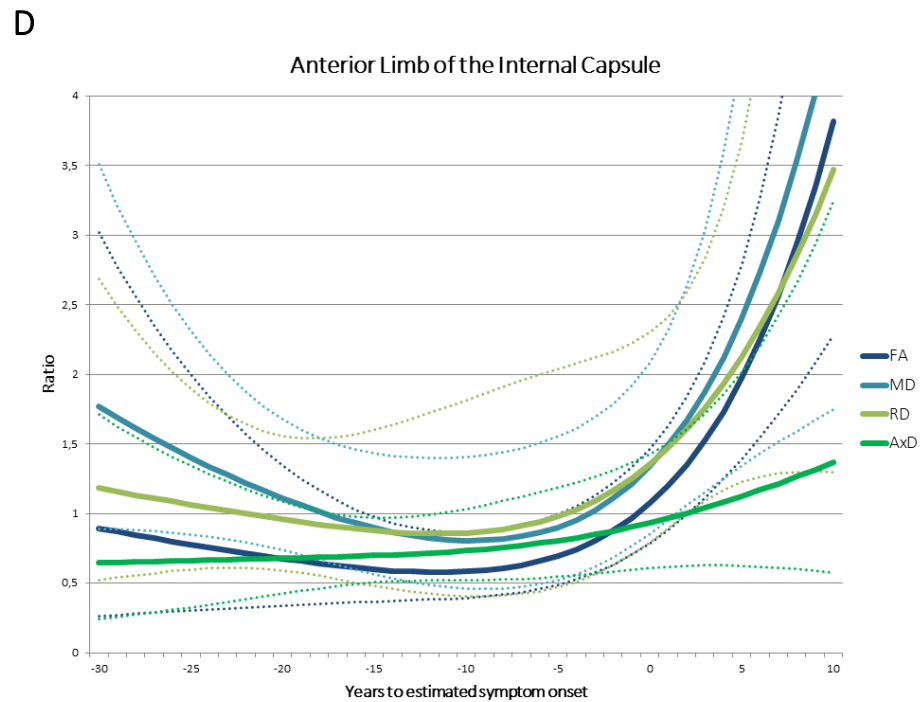

Supplement: Supplementary file 6 — Data S6. Ratio values across estimated years to symptom onset in GRN mutation carriers versus noncarriers. [file ACN3-5-1025-s006.pdf]
